# Endometrial gland-specific progestagen-associated endometrial protein and cilia gene splicing changes in recurrent pregnancy loss

**DOI:** 10.1530/RAF-22-0002

**Published:** 2022-08-15

**Authors:** Jennifer E Pearson-Farr, Gabrielle Wheway, Maaike S A Jongen, Patricia Goggin, Rohan M Lewis, Ying Cheong, Jane K Cleal

**Affiliations:** 1 Human Development and Health, University of Southampton, Faculty of Medicine, Southampton, UK; 2 Complete Fertility Centre Southampton, Princess Anne Hospital, Division of Women and Newborn, Southampton, UK; 3 Biomedical Imaging Unit, Faculty of Medicine, University of Southampton, Southampton, UK

**Keywords:** fertility, gland secretions, exon skipping, gene transcription, uterine environment

## Abstract

Endometrial glands are essential for fertility, consisting of ciliated and secretory cells that facilitate a suitable uterine environment for embryo implantation. This study sought to determine whether an endometrial gland-specific transcriptome and splicing profile are altered in women with recurrent pregnancy loss. Our data provide a comprehensive catalogue of cilia and progestagen-associated endometrial protein (*PAEP*) gene isoforms and relative exon usage in endometrial glands. We report a previously unannotated endometrial gland cilia transcript *GALNT11* and its susceptibility to exon skipping. Key endometrial receptivity gene transcripts are also reported to change in endometrial glands of women with recurrent pregnancy loss. The endometrial gland cilia and PAEP targets identified in this study could be used to identify a perturbed endometrium, isolate causes of recurrent pregnancy loss and develop targeted therapies in personalised medicine.

**Lay summary:**

Successful embryo implantation is a trade-off between the lining of the womb which receives an implanting embryo, termed the endometrium, and a good quality embryo. For days 21–24 of the menstrual cycle, the endometrium undergoes changes into a receptive state in which it can receive an implanting embryo. Inappropriate endometrial receptivity is thought to underlie recurrent pregnancy loss. Improving pregnancy success in women with recurrent pregnancy loss requires an increased understanding of the endometrium at the molecular level. Genes contain the instructions for the cell and which genes are turned on or off determine how well it can do its role. We sought to determine a gene expression pattern of human endometrial glands in women with recurrent pregnancy loss (*n* = 5) vs a control group (*n* = 5). We identify target genes altered in women with recurrent pregnancy loss. Endometrial gland markers could be used to identify inappropriate endometrial receptivity.

## Introduction

The endometrium is the lining of the womb in which the embryo implants at the start of pregnancy. The regenerated endometrium becomes receptive to implantation in the period following ovulation termed the implantation window (7–10 days after the luteinising hormone (LH) surge; LH+7–10). Inappropriate receptivity is thought to underlie reproductive failure in women with recurrent pregnancy loss ≥3 miscarriages), subfertility (>12 months of inability to conceive) and recurrent implantation failure (failure of ≥2 *in vitro* fertilisation (IVF) cycles with 3 good-quality blastocysts replaced). A less-receptive endometrium is implicated in subfertility and recurrent implantation failure, as implantation fails in over 50% of IVF patients despite the selection of good-quality embryos. Whereas an over-receptive endometrium may underlie recurrent pregnancy loss by allowing the implantation of low-quality embryos which increases the risk of pregnancy loss (Macklon & Brosens 2014). The genetics and cell biology of endometrial receptivity remain poorly understood, limiting the scope for clinical intervention in the 2% of women who suffer from unexplained recurrent pregnancy loss (Ford & Schust 2009).

Studies have attempted to establish molecular markers of endometrial receptivity, yet to date, no consistent panel of genes has been identified. Microarray analysis has identified a potential gene expression profile, termed the endometrial receptivity array (ERA) (Díaz-Gimeno *et al.* 2011). Analysis using this ERA does suggest a non-receptive endometrium underlies implantation failure (Ruiz-Alonso *et al.* 2013). A qPCR-based panel of selected genes to predict endometrial receptivity has also been developed (Enciso *et al.* 2018), but again this is not whole genome-based. Unfortunately, despite a large amount of data supporting endometrial biomarkers established by omics, there is still little evidence to link omics data to pregnancy outcomes (Hernández-Vargas *et al.* 2020).

Attempts to isolate causes of reproductive failure have been challenged by the cellular heterogeneity of endometrial biopsies (Hu *et al.* 2014, Suhorutshenko *et al.* 2018). To date, few studies account for the contribution of different cell types which is important for the overall gene expression profile of the endometrium (Garcia-Alonso *et al.* 2021). To address this, further studies need to be carried out on specific populations of cells within the receptive endometrium in order to understand patterns of gene expression in different cells and tissues. Furthermore, to enable early diagnosis in patient care, more work is required to establish a transcriptomic profile of the endometrium from women with a lower order of miscarriages, less distinct than a high order of miscarriages (Craciunas *et al.* 2021).

Endometrial glands play an essential role in supporting the uterine environment for successful embryo implantation, conceptus development and placentation. Reproductive failure is associated with endometrial gland loss in mouse and sheep gene knock-out models (Gray *et al.* 2002, Filant & Spencer 2013), reinforcing the importance of these glandular cell types and their secretions in successful pregnancy. Endometrial gland-specific transcriptomic differences are reported in cases of endometriosis (Suda *et al.* 2018), yet the endometrial gland-specific transcriptome has not been investigated in recurrent pregnancy loss. In order to address the gaps in the literature regarding cell-specific expression in different parts of the endometrium and delineate gene expression in this study, we carried out whole transcriptome RNAseq analysis of endometrial glands from women with recurrent pregnancy loss.

## Methods

### Study participants

Participants were recruited for the collection of an endometrial biopsy at a tertiary fertility and gynaecology referral centre in Southampton. Recruited participants met study criteria including aged 21–37 years, no hormonal contraception, no infections and no uterine pathologies. Two participant groups included control participants and recurrent pregnancy loss participants. Control participants (*n* = 5) were recruited from fertile women who elected to donate eggs having met the criteria for egg donation, while an exclusion criteria for control participants was a history of pregnancy loss. Recurrent pregnancy loss participants (*n* = 5) had a history of three or more first trimester losses (RCOG 2011). Samples were collected in natural cycles, and no participants were hormone primed. Control and recurrent pregnancy loss participants were matched by the day of the menstrual cycle to form five pairs. Informed written consent was given by all participants, and ethical approval for this study was given by the Isle of Wight, Portsmouth & South East Hampshire Research Ethics Committee (08/H0502/162). Endometrial biopsies were collected using a Pipelle catheter (Stocker *et al.* 2017) during the window of implantation (LH+4–10) and immediately immersed into 50:50 Dulbecco's modified Eagle medium (DMEM)/Ham’s F12 nutrient mixture, containing 5% streptomycin for endometrial gland isolation.

### Endometrial gland isolation and RNA preparation

Endometrial gland isolation was performed by enzyme digestion within 1 h of tissue collection. Endometrial tissue pieces were minced into smaller pieces before being digested with 0.7 mg/mL type 1A collagenase in 50:50 DMEM/Ham’s F12 nutrient mixture, containing 5% streptomycin at 37°C for 2× 15 min intervals with gentle agitation. The digested cell suspension was then passed through a serum gradient to isolate the endometrial gland fraction of the population. The isolated endometrial gland fraction was then passed through a 50 µm sieve to remove other endometrial cell types. Endometrial glands were then stored in 700 µL QIAol lysis reagent at −80°C until RNA extraction. RNA extraction was carried out using the Qiagen miRNeasy extraction kit. The RNA yield was quantified by Thermo Scientific Nanodrop 1000 spectrophotometer and RNA quality was analysed using an RNA Nano chip on an Agilent 2100 Bioanalyser (RNA integrity numbers: C1 = 9.4, C2 = 9.2, C3 = 9.5, C4 = 9.4, C5 = 8.9 , RPL1 = 7.7, RPL2 = 9.5, RPL3 = 9.0, RPL4 = 9.4, RPL5 = 8.8).

### Library preparation and RNA sequencing

Library preparation was performed using the TruSeq Stranded mRNA Library Prep kit (Illumina). The final library was quantified by a Roche KAPA library quantification kit (Illumina) and by the Agilent 2100 Bioanalyser. Paired-end RNA sequencing (2 × 150 bp) was carried out on an Illumina NextSeq 550.

### Serial block face scanning electron microscopy

Endometrial tissue pieces from a control participant at the implantation window were fixed in 3% glutaraldehyde 0.1 M sodium cacodylate buffer at pH 7.4, stained with heavy metals and dehydrated (Goggin *et al.* 2020). The endometrial pieces were polymerised in Spurr resin at 60°C for 16+ h. The resin block was trimmed to a frustum with a top face approximately 500 µm^2^ including a gland. This sub-block was mounted onto an aluminium pin with conductive glue and sputter coated with gold/palladium. The endometrial gland was imaged by Gatan 3View inside an FEI Quanta 250 FEGSEM microscope at 3.0 kV accelerating voltage and a vacuum of 40 Pa (Palaiologou *et al.* 2020). A stack of consecutive images were generated at a constant voxel size of 0.01 × 0.01 × 0.05 µm. Segmentation and reconstruction were carried out using Amira and Fiji Image J.

### Transmission electron microscopy

Endometrial tissue pieces were fixed in 3% glutaraldehyde 0.1 M sodium cacodylate buffer at pH 7.4, stained with heavy metals and dehydrated using a graded ethanol series (Palaiologou *et al.* 2020). The samples were polymerised and encapsulated in Agar low viscosity resin at 60°C for 16+ h. Thin sections (90 nm) were cut, stained with lead citrate and imaged using Hitachi HT7700 TEM at 100 kV.

## Quantification and statistical analysis

### Differential gene expression analysis

Raw FASTQ reads were aligned to the human genome via STAR 2.7.3a alignment using human genome 38 and trimmed with Trimmomatic. Quality control was assessed by FastQC v0.11.3. Gene count data were normalised, and paired differential gene expression analysis was carried out in RStudio R-4.0.3 package DESeq2 v1.30.1 (Love *et al.* 2014). The Empirical Bayes approach to false discovery rate (FDR) was applied to correct for multiple testing at 5%. Significance was determined by a Wald test and accepted as *P* ≤ 0.05. To increase stringency, a further log fold-change threshold of 1.15 was applied.

### Gene ontology functional enrichment

Genes which were significantly differentially expressed in the endometrial glands of recurrent pregnancy loss patients compared to controls (no log fold-change threshold) were mapped to pathways using the publicly available software Toppgene (Division of Bioinformatics, Cincinnati Children’s Hospital Medical Centre). A B&H FDR was used to correct findings *P* < 0.05.

### Differential gene transcript expression analysis and splicing analysis

Raw FASTQ reads underwent adapter trimming and quality filtering (reads containing *N* > 10%, reads where >50% of read has Qscore ≤ 5). Paired FASTQ files were aligned to GRCh38 human genome reference using GENCODE v29 gene annotations (Frankish *et al.* 2019) and STAR v2.6.0a splice aware aligner (Dobin *et al.* 2013), using ENCODE recommend options ((https://github.com/alexdobin/STAR/blob/master/doc/STARmanual.pdf); 3.2.2 in the STAR manual). The two-pass alignment method was used, with soft clipping activated.

### Alignment quality control

Binary sequence alignment map format (BAM) files sorted by chromosomal coordinates assessed for saturation of known splice junctions were calculated using RSeqQC v3.0.1 (Wang *et al.* 2012).

### Alignment to reference transcriptome and transcript level abundance estimates

Salmon tool was used to perform transcript abundance estimates from raw FASTQ files using selective alignment with a decoy-aware transcriptome built from GRCh38 (Patro *et al.* 2017).

### Differential splicing analysis

The computational tool 'RNA sequence data Multivariate Analysis of Transcript Splicing r(MATS)' v4.0.2 (rMATS turbo) was used to statistically measure differences in splicing between replicates of WT and mutant sequence (Shen *et al.* 2014). BAM files aligned with STAR v2.6.0a two-pass method with soft clipping suppressed were used as input.

## Results

**Figure 1:**
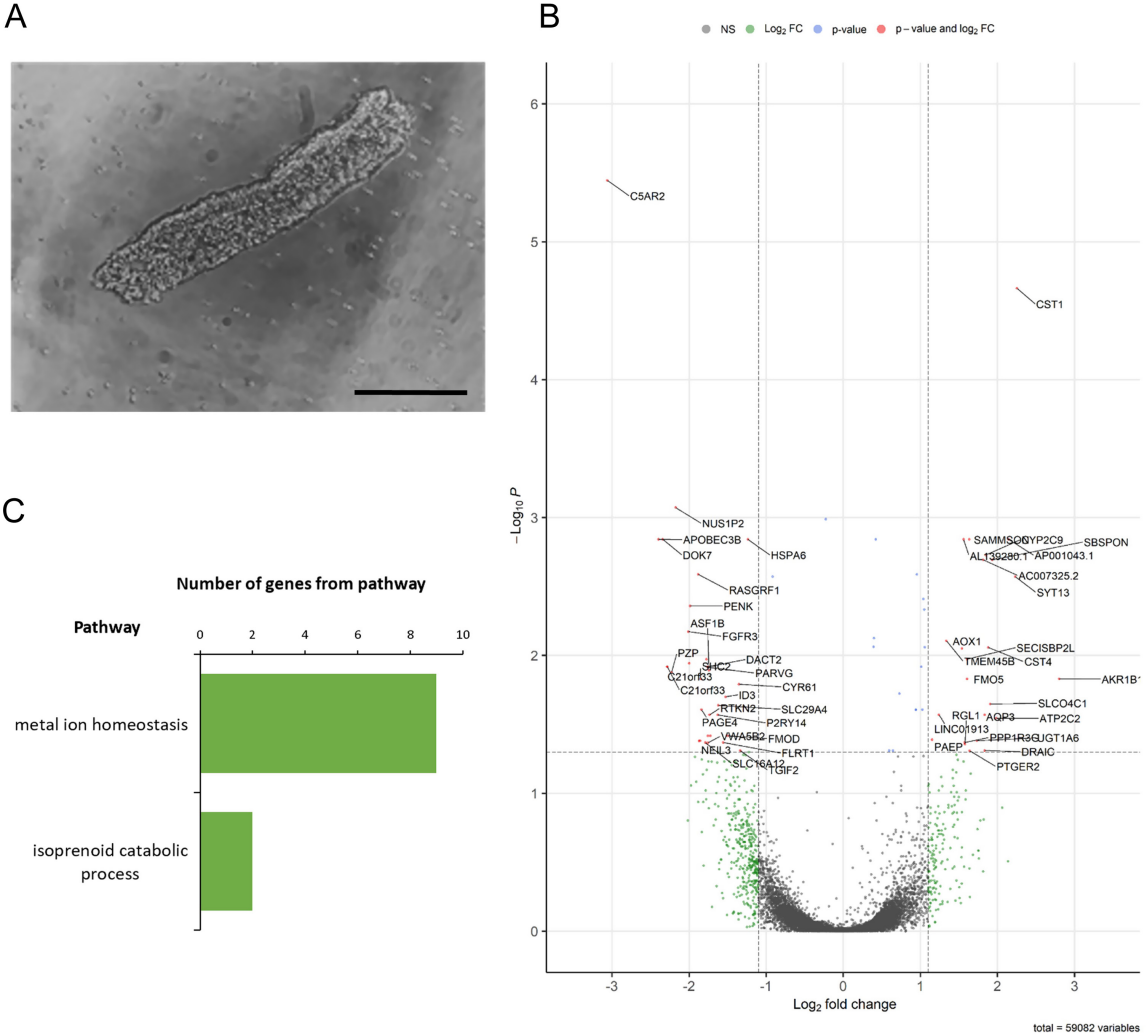
Altered endometrial gland gene expression in women with recurrent pregnancy loss. (A) Image of isolated endometrial gland (scale bar = 50 µm) captured using light microscopy. (B) Volcano plot representing differentially expressed genes in endometrial glands from women with recurrent pregnancy loss (RPL, *n* = 5) vs controls (C, *n* = 5), fold difference between log_2_ normalised expression plotted vs −log_10_ adjusted *P* value. (C) Biological processes containing differentially expressed genes in endometrial glands from women with recurrent pregnancy loss following analysis of all differentially expressed genes. FDR B&H corrected q value < 0.05.

To investigate differential gene and transcript expression and differential splicing in the endometrial glands of women with recurrent pregnancy loss compared to controls, we performed paired-end 2 × 150 bp RNA sequencing to an average depth of 24.7 million reads per sample on 5 pairs of isolated endometrial glands from recurrent pregnancy loss patients vs controls matched by the day of the menstrual cycle (Fig. 1A and Table 1). Seventy-three genes were differentially expressed using a 5% FDR in the glandular epithelium from women with recurrent pregnancy loss vs controls (Fig. 1B). Of these, 38 genes were upregulated and 35 genes were downregulated in recurrent pregnancy loss. Fifty-seven genes met a more stringent 1.15 log fold-change threshold, and of these, 24 genes were upregulated and 33 genes were downregulated (Fig. 1B). Differential gene expression in the glandular epithelium from women with recurrent pregnancy loss compared to controls included upregulation of the glandular secretory product genes progestagen-associated endometrial protein (*PAEP*) and *SYT13* involved in transport vesicle docking to the plasma membrane. Significantly enriched biological processes in the glandular epithelium from women with recurrent pregnancy loss included metal ion homeostasis and isoprenoid catabolic processes (Fig. 1C). Unsupervised clustering principal component analysis reported that cycle length clustered at day 28 of the menstrual cycle and therefore was included as a confounding factor for differential gene expression analysis (Supplementary Fig. 1, see section on supplementary materials given at the end of this article).

**Table 1:** Participant clinical characteristics. Data are presented as mean (s.d.).

Characteristics	Control (*n* = 5)	Recurrent pregnancy loss (*n* = 5)
Demographic characteristics		
Age (years)	28.8 (5.1)	33.0 (3.9)
BMI	22.0 (2.2)	24.2 (3.0)
Menstrual cycle characteristics		
Day of menstrual cycle	21.0 (2.0)	21.4 (1.9)
Length of menstrual cycle (days)	28.8 (0.9)	27.8 (1.9)
Fertility history		
Contraceptive use in last year	None	None
Number of pregnancies	1 (1)	7 (2) *
Number of miscarriages	0 (0)	6 (3) *
Met AMH criteria for egg donation	Yes	n/a

AMH, anti-Mullerian hormone; n/a, not applicable.

*Significantly different from control group indicated by *P < 0.01.

**Figure 2:**
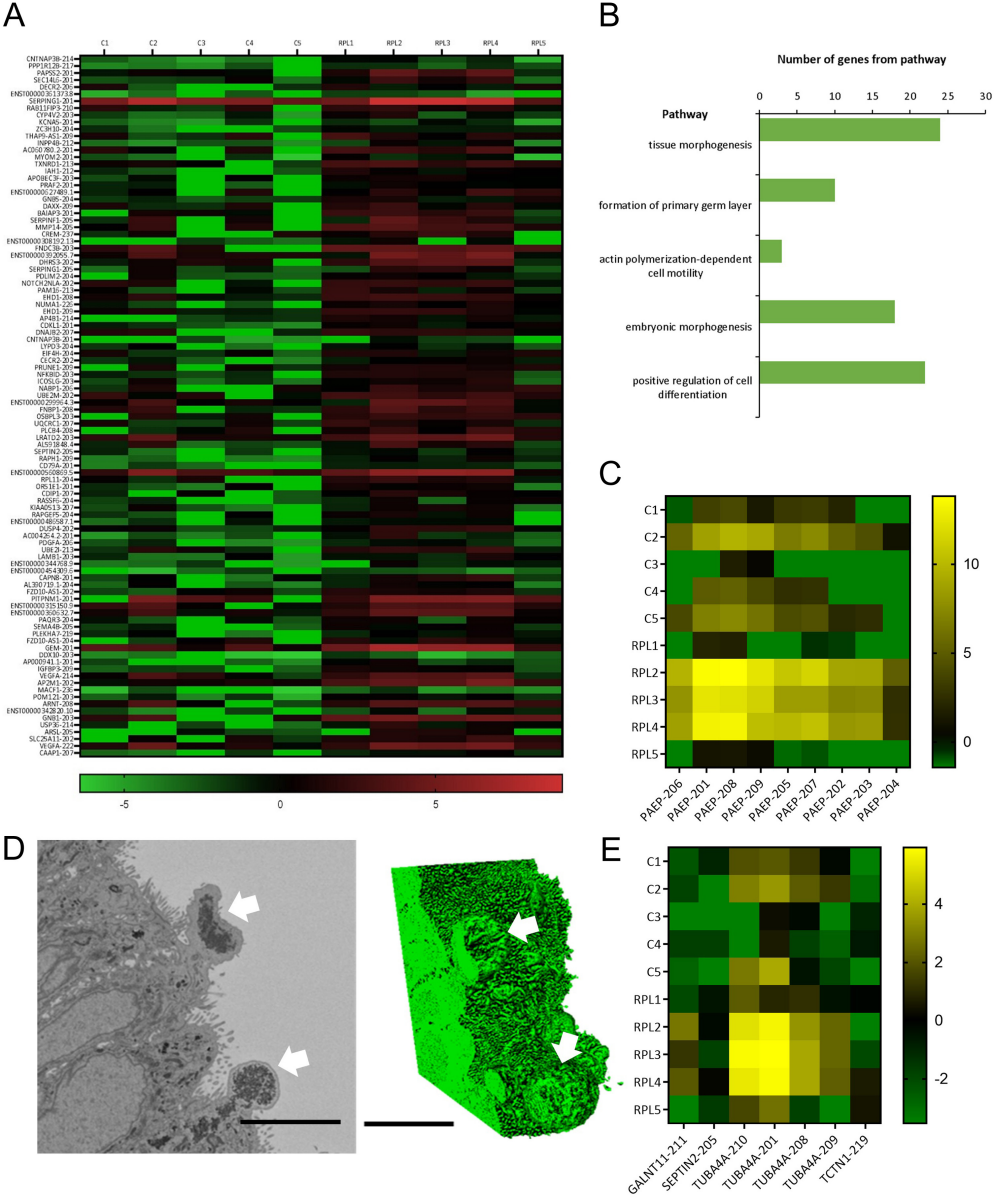
Altered endometrial gland gene transcript expression in women with recurrent pregnancy loss. (A) Heat map representing top 100 differentially expressed gene transcripts in endometrial glands from women with recurrent pregnancy loss (RPL, *n* = 5) vs controls (C, *n* = 5), data presented as log_2_. (B) Biological processes containing differentially expressed gene transcripts in endometrial glands from women with recurrent pregnancy loss following analysis of all differentially expressed genes. FDR B&H corrected q value < 0.05. (C) Heat map representing altered PAEP gene transcript expression in endometrial glands in recurrent pregnancy loss vs controls. (D) TEM image and 3D reconstruction of electron-dense material budding from the apical surface of the glandular epithelium in a control participant (white arrows), scale bar = 5 µm. (E) Heat map representing altered cilia gene transcript expression in endometrial glands in recurrent pregnancy loss vs controls.

To further investigate transcriptomic differences between the endometrial glands of women with recurrent pregnancy loss and controls, we carried out transcript-level expression analysis and alternative splicing analysis on our RNAseq data. Two-hundred and seventy-eight differentially expressed gene transcripts were reported in the glandular epithelium of women with recurrent pregnancy loss vs controls. Of those, 257 gene transcripts were upregulated and 21 gene transcripts were downregulated in recurrent pregnancy loss (Fig. 2A). Specific gene transcripts included a significant upregulation of pre-identified glandular secretory product *MUC16-204*, glandular progenitor cell marker *LRIG1-205*, intraciliary transport particle *IFT122-204* and known endometrial receptivity marker *LAMB3* (*LAMB-201*, *LAMB-204* and *LAMB3-203*). The recurrent pregnancy loss group is a heterogeneous group. The two most significantly enriched biological processes in the glandular epithelium of women with recurrent pregnancy loss include tissue morphogenesis and positive regulation of cell differentiation (Fig. 2B).

**Figure 3:**
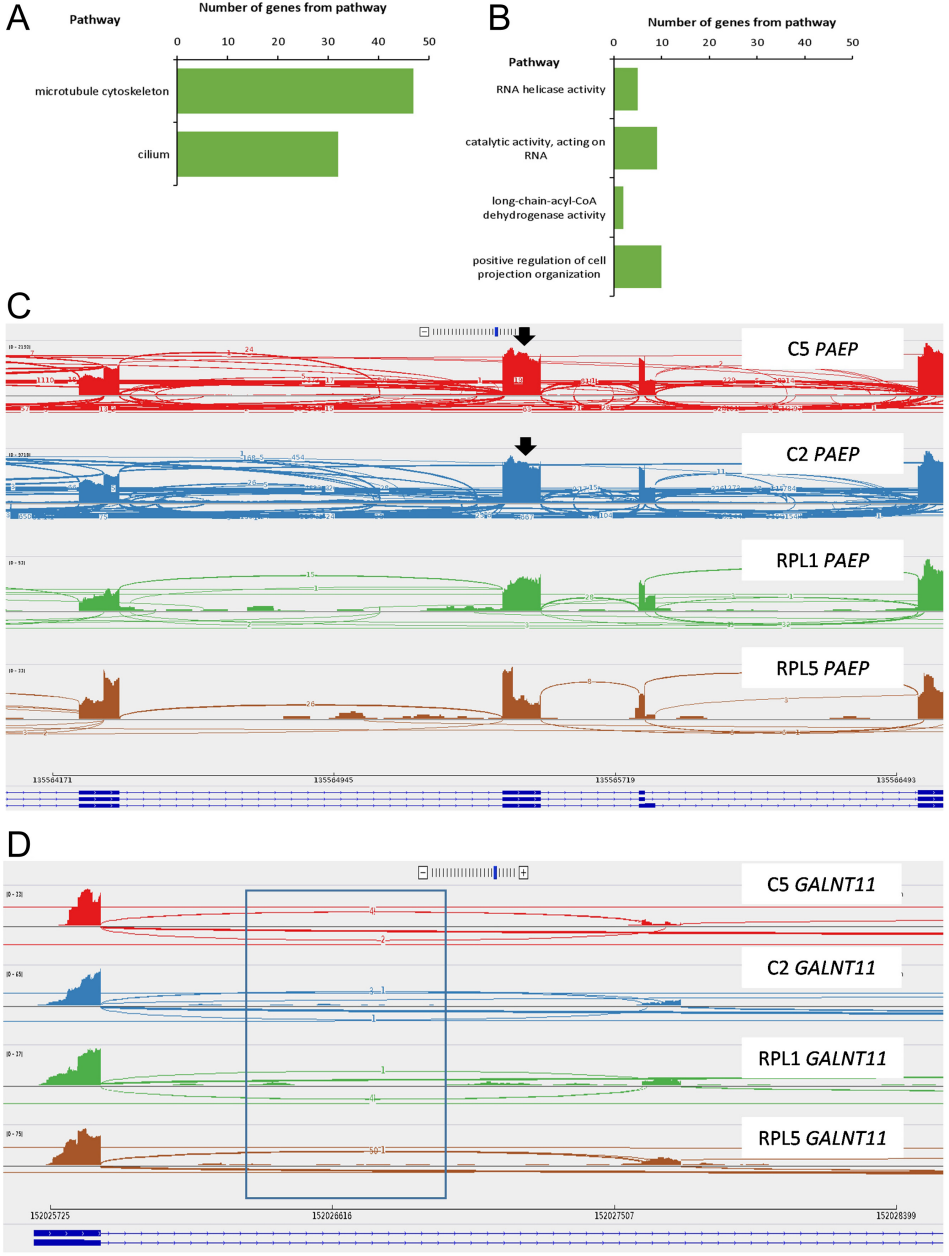
Alternative splicing events in endometrial glands in women with recurrent pregnancy loss. (A) Cellular components commonly undergoing exon skipping in the endometrial glands of women with recurrent pregnancy loss (*n* = 5) compared to controls (*n* = 5). FDR B&H corrected q value < 0.05. (B) Molecular functions and biological processes commonly undergoing intron retention in the endometrial glands of women with recurrent pregnancy loss compared to controls. FDR B&H corrected q value. (C) Sashimi plots of differentially spliced *PAEP* demonstrating a decline in exon skipping events in recurrent pregnancy loss (RPL; green and brown) compared to controls (C; red and blue; black arrows highlight exon skipping events). Each plot shows gene expression (bar graph), the number of reads split across the splice junction (curved lines), exons (blue bar at the bottom of the plot) and introns of the corresponding gene (dotted lines at the bottom of the plot). (D) Sashimi plot demonstrating increased reads split across splice junctions in GALNT11 in women with recurrent pregnancy loss compared to controls.

Alternative splicing events were significantly different in the glandular epithelium from women with recurrent pregnancy loss vs controls. These included exon skipping, intron retention, mutually exclusive exons, alternative 3’ splice site and alternative 5’ splice site. Four hundred and eighty-five gene transcripts were significantly altered in exon skipping (<0.05 FDR) and were enriched in the cilium and microtubule skeleton cellular components (Fig. 3A). Specific human gene transcripts included those involved in ciliary function (*GALNT11*, *FBXL13* and *LRRC6*). A previously unannotated *GALNT11* transcript was reported (*GALNT11-211*); although *GALNT11* is expressed at low levels, there was a significant splicing difference between the numbers of reads spanning the exons (Fig. 3D). *GALNT11* exon skipped starting at coordinate 152027596 and ending at coordinate 152027718. Other cilia gene transcripts present include *SEPTIN2-205*, *TUBA4A-210*, *TUBA4A-201*, *TUBA4A-208*, *TUBA4A-209* and *TCTN1-219* (Fig. 2E). *GAS5* and *DYNLL1* also had significantly altered intron retention events.

Eighty-three gene transcripts were significantly changed in intron retention (<0.05 FDR) and were enriched in gene pathways from biological processes termed: RNA helicase activity, catalytic activity acting on RNA, long chain dehydrogenase activity and positive regulation of projection organisation (Fig. 3B). Eighty-six gene transcripts had significantly altered mutually exclusive exons in the glandular epithelium of women with recurrent pregnancy loss compared to controls (<0.05 FDR). Seventy gene transcripts had significantly alternative 3’ splice sites in the glandular epithelium of women with recurrent pregnancy loss compared to controls (<0.05 FDR). Finally, 49 gene transcripts had significantly alternative 5’ splice sites in the glandular epithelium from women with recurrent pregnancy loss compared to controls (<0.05 FDR).

**Figure 4:**
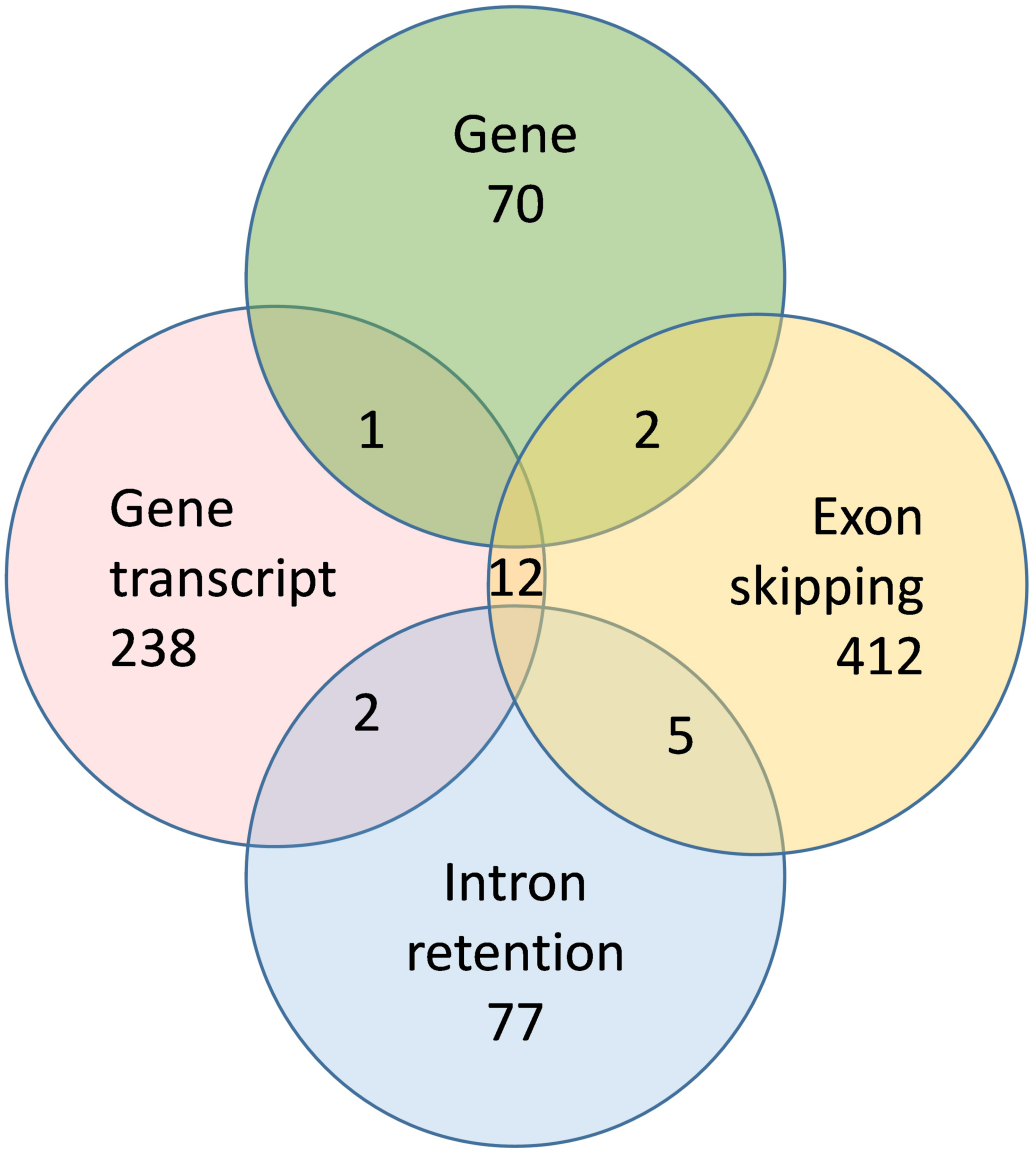
Common genes differentially expressed, at the gene level, the gene transcript level and alternative splicing events. The number of genes expressed as count data.

Seven *PAEP* gene transcripts were upregulated in endometrial glands from women with recurrent pregnancy loss compared to controls. These included *PAEP-206*, *PAEP-201*, *PAEP-208*, *PAEP-209*, *PAEP-205*, *PAEP-207* and *PAEP-202* (Fig. 2C). Alternative splicing of* PAEP* in endometrial glands of women with recurrent pregnancy loss vs controls (<0.05 FDR) demonstrated cryptic splice site usage in *PAEP* (Fig. 3C). Three *PAEP* exon skipping events were significantly altered in recurrent pregnancy loss vs controls. Exons skipped included *PAEP* start coordinate 135562359 and end coordinate 135562433, *PAEP* 135562293–135562433, and *PAEP* 135564243–135564354. *PAEP* intron retention events were not significantly changed in the glandular epithelium of women with recurrent pregnancy loss vs controls. Five mutually exclusive exon *PAEP* events were significantly different in glandular epithelium from women with recurrent pregnancy loss vs controls (<0.05 FDR; Table 2). Two *PAEP* alternative 3’ splice sites were significantly altered in the glandular epithelium from women with recurrent pregnancy loss compared to controls. No alternative *PAEP* 5’ splice sites, however, were altered in the glandular epithelium from women with recurrent pregnancy loss compared to controls. When comparing the transcriptome between women with recurrent pregnancy loss to controls, 12 genes were common to differential gene expression, differential gene transcript expression and alternative splicing events (Fig. 4). TEM imaging in endometrial gland secretory cells from control participants demonstrated structures containing electron-dense material budding from the apical surface of the epithelium. Serial block-face scanning electron microscopy (SBF- SEM) reconstructions show that these were approximately spherical in shape (Fig. 2D).

**Table 2:** Mutually exclusive exon events for PAEP significantly altered in the glandular epithelium of women with recurrent pregnancy loss compared to controls.

	First exon		Second exon	
	Start coordinate	End coordinate	Start coordinate	End coordinate
PAEP	135565413	135565514	135565784	135565830
PAEP	135562819	135562893	135565409	135565514
PAEP	135562819	135562893	135565337	135565514
PAEP	135562819	135562893	135565413	135565514
PAEP	135565337	135565514	135565784	135565830

## Discussion

Here we show that endometrial glandular ciliated and secretory cells exhibit dysregulation in women with recurrent pregnancy loss vs controls. Differential transcript expression and relative exon usage are reported, identifying novel *PAEP* and *GALNT11* transcripts produced through the use of cryptic splice sites. Novel cilia and secretory RNA targets identified in this study may pose new avenues for therapeutics for better reproductive outcomes.

We demonstrate upregulation of the glandular secretory *PAEP* gene in women with recurrent pregnancy loss, and that this gene is differentially spliced in women with recurrent pregnancy loss. As well as showing overall increased *PAEP* gene expression, women with recurrent pregnancy loss express novel *PAEP* transcripts produced through the use of cryptic splice sites. *PAEP* upregulation has been reported by other studies (Burmenskaya *et al.* 2017), and alternative *PAEP* splicing has previously been found in the female reproductive tract (Garde *et al.* 1991), but our data provide further detail on the specific *PAEP* transcripts expressed in the endometrial glands and the presence of novel transcripts produced through cryptic splice site usage in women with recurrent pregnancy loss. This may represent a novel RNA target for therapeutic intervention in recurrent pregnancy loss. Splice-switching oligonucleotides (SSOs) and other antisense oligonucleotides (ASOs) are becoming increasingly used for the treatment of a range of genetic conditions and cancers, and so identification of RNA transcripts which can be targeted with ASOs and SSOs is a very promising avenue for treatment (Dhuri *et al.* 2020). In addition, our electron microscopy data are indicative of microparticle secretion from the glandular epithelial cells, reinforcing their importance in facilitating the endometrial environment.

Post-translational changes to PAEP have been associated with endometrial cancer, supporting evidence that altered PAEP has an impact on endometrial function (Hautala *et al.* 2020). Since a recent manuscript has shown that PAEP modulates cytotoxic uterine natural killer cells which pose an adverse effect on the growing fetus and subsequent pregnancy loss, it will be important to perform further studies to investigate and characterise the different PAEP isoforms in recurrent pregnancy loss, relevant splicing profiles and if these events are occurring independently (Dixit & Karande 2020). PAEP is a major progesterone-regulated glycoprotein. Changes to progesterone receptors in the glandular epithelium could be influenced by a decrease in TGIF2 expression, thereby preventing the repression of TGF beta-responsive genes (Snijders *et al.* 1992). This could be one factor that accounts for cycle length variation seen in the RNA-sequencing data.

The enrichment of differentially spliced cilia gene transcripts, including a novel transcript of *GALNT11*, in women with recurrent pregnancy loss may offer novel insights into the role of endometrial gland cilia in female fertility. The role of cilia in women’s fertility is a poorly understood area, with most work focussing on Fallopian tube cilia function and dysfunction. Although little is known about the role of cilia in endometrial function, a recent single-cell study has identified both ciliated and non-ciliated epithelial cell populations (Garcia-Alonso *et al.* 2021). Transcriptomic studies have also highlighted a potential relevance for cilia function in the endometrium of women with endometriosis and that of women over 35 years (Devesa-Peiro *et al.* 2020, 2022). Variants in genes linked to primary ciliary dyskinesia (DNAH11 and CCNO) are mutated in women with infertility, and gene variants identified from women with recurrent pregnancy loss were also related to cilia motility disorders (Qiao *et al.* 2016, Maddirevula *et al.* 2020).

Studies in this area are confused by the fact that atypical cilia are commonly observed in the human endometrial epithelium as an artefact of the relatively high turnover rate of this tissue caused by menstruation (Denholm & More 1980). Furthermore, ciliary beat frequency in the Fallopian tube, often used as a proxy for ciliary health and function, varies in relation to the stage of menstrual cycle and anatomical location (Lyons *et al.* 2002) and is affected by environmental factors such as cigarette smoking (Magers *et al.* 1995), steroids (Mahmood *et al.* 1998) and reproductive tract infections (Mardh *et al.* 1976, 1979, McGee *et al.* 1981). Studies of female fertility in inherited genetic conditions associated with cilia (ciliopathies), such as primary ciliary dyskinesia (PCD), have largely been contradictory and inconclusive (Blyth & Wellesley 2008, Raidt *et al.* 2015, Goutaki *et al.* 2016). A recent comprehensive study of fertility in males and females with PCD, however, found that women with PCD reported infertility at a higher rate than the general population but a miscarriage rate lower than the general population (Vanaken *et al.* 2017). This study suggested that mutations in specific cilia genes lead to female infertility, and our work further suggests that other cilia genes may be differentially spliced in recurrent pregnancy loss, suggesting a complex role for cilia in female fertility. We recognise that a limitation of our study is the small sample size; however, our endometrial gland-specific approach compared to whole endometrium (Labarta *et al.* 2021) is unique, and a larger cohort study and validation work should be performed.

## Conclusions

Our data provide a detailed transcriptomic profile of gland-specific differences in recurrent pregnancy loss, leading to a more accurate account of where the gene expression changes are occurring. Our transcriptome data provide a comprehensive catalogue of gland-specific target genes, transcripts and splice variants that are altered in recurrent pregnancy loss. Future work should aim to make investigations on isolated endometrial cell populations to account for cell heterogeneity, which will build evidence for therapeutic targets. The development of endometrial organoid culture methods (Abbas *et al.* 2020) may be a powerful tool to support studies of molecular mechanisms alongside the study of clinical patient samples.

## Supplementary Material

Supplementary Figure 1: Principle component analysis of cycle length.

## Declaration of interest

G W is employed by Illumina Inc. Ying Cheong is a Associate Editor of *Reproduction and Fertility.* Ying Cheong was not involved in the review or editorial process for this paper, on which she is listed as an author. All other authors report no conflicts of interest.

## Funding

This project was funded by Wellbeing of Women (RG2147). Equipment in the Biomedical Imaging Unit was supported by MR/L012626/1 Southampton Imaging under MRC UKRMP.

## Data availability

Data discussed in this study have been deposited on the NCBI’s Gene Expression Omnibus (Edgar *et al.* 2002) and are available through the GEO Series accession number GSE GSE183555.

## Ethics approval

Isle of Wight, Portsmouth & South East Hampshire Research Ethics Committee (08/H0502/162).

## Consent to participate

Informed written consent was given by all participants.

## Consent for publication

Participant information was anonymised.

## Author contribution statement

J P and J S performed sample collection and laboratory analysis. G W, J S and J P performed analysis of transcriptomic data. All authors contributed interpretation all data and the writing of the study. J C, Y C and R L initiated, designed and obtained funding for the study.
