# Supplementary figures and images for: Endometrial gland-specific progestagen-associated endometrial protein and cilia gene splicing changes in recurrent pregnancy loss
Source: Reprod Fertil. 2022 Aug 15;3(3):162–72. doi: 10.1530/RAF-22-0002 (PMC9513660; doi:10.1530/RAF-22-0002)

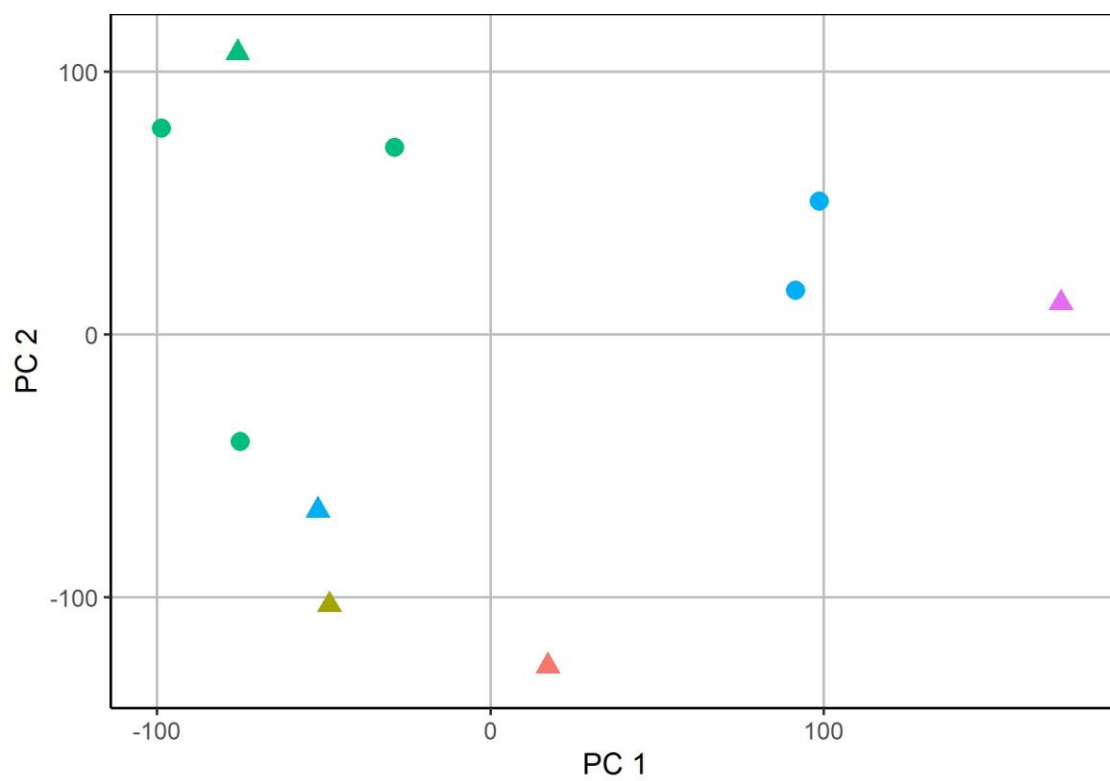

Length of the Cycle:

|    |    |           |
|----|----|-----------|
| 26 | 28 | irregular |
| 27 | 30 |           |

Supplement: Supplementary Figure 1: Principle component analysis of cycle length. [file supplementary_figure_1.pdf]
